# Presence of Methicillin Resistant *Staphylococcus aureus* (MRSA) in Feces of the Small Indian Mongooses (*Urva auropunctata*) on Saint Kitts and Nevis, West Indies

**DOI:** 10.3390/antibiotics11080990

**Published:** 2022-07-22

**Authors:** Andreas Hoefer, Anne A. M. J. Becker, Arshnee Moodley, Filip Boyen, Patrick Butaye

**Affiliations:** 1 School of Veterinary Medicine, Ross University, Basseterre P.O. Box 334, Saint Kitts and Nevis; andreashoefer@hotmail.com (A.H.); abecker@rossvet.edu.kn (A.A.M.J.B.); 2 International Livestock Research Institute, Animal and Human Health, Nairobi 00100, Kenya; a.moodley@cgiar.org; 3 Department of Veterinary and Animal Sciences, University of Copenhagen, 1817 Frederiksberg, Denmark; 4 Department of Pathobiology, Pharmacology and Wildlife Medicine, Faculty of Veterinary Medicine, Ghent University, 9820 Merelbeke, Belgium; filip.boyen@ugent.be

**Keywords:** MRSA, wildlife, *Staphylococcus aureus*, whole genome sequencing, One Health

## Abstract

Although, historically, Methicillin-Resistant *Staphylococcus aureus* (MRSA) was restricted to humans, since 2005 these strains emerged in livestock and wildlife. Therefore, a One Health approach was applied to analyze the diversity and characteristics of *S. aureus* strains isolated from the invasive species of mongoose (*Urva auropunctata*) in St. Kitts. Fecal samples collected from these animals (*n* = 81) were cultured on selective agar. The isolated *S. aureus* strains were identified using MALDI-TOF and further characterized by whole genome sequence analysis. The fecal microbiome study identified the presence of *S. aureus* in 5 animals. Both MSSA (*n* = 3) and MRSA (*n* = 2) strains were identified. The two MRSA isolated were nearly identical ST5 SCC*mec* IVa (2B) strains. The two MSSA isolated were a new ST7434, pertaining to clonal complex 30, and the other belonged to ST5, but unrelated to the MRSA ST5. The SCC*mec* IVa (2B) is, however, the main SCC*mec* in human MRSA of different STs identified in St Kitts, indicating potential horizontal transmission events. In conclusion, a new type of MSSA, ST7434, was found and MRSA ST5 t002 SCC*mec* IVa (2B) found its way into wildlife on a small Caribbean Island. Further One Health studies are necessary to determine the role of MRSA in wildlife.

## 1. Introduction

Decades after the emergence of Methicillin resistant *Staphylococcus aureus* (MRSA) in humans, first in health care centers and then also in the community, livestock associated MRSA (LA-MRSA) were discovered. Marked geographical differences were found in the prevalence of LA-MRSA, with the European strains differing significantly from Asian and North American strains [1]. The body of evidence that MRSA has also emerged in wildlife is ever increasing. A recent review showed the importance of the wildlife reservoir of MRSA and potential sentinels for antimicrobial resistance and the Clonal Complexes (CCs) 398 and 130 were identified as the most prevalent with an over-representation of the *mecC* gene [2]. However, it should be pointed out that there are few studies available, and those may represent a bias towards Europe as most studies were performed in this region.

The small Indian mongoose was first introduced into the Caribbean in the 1800s to control rats and snakes on sugar cane plantations and are regarded as an invasive species. While the rats could not be controlled, the snakes disappeared from the island and now the mongooses endanger mainly birds nesting on the ground.

St Kitts and Nevis are two small Caribbean islands with approximately 57,000 inhabitants. We recently observed a high prevalence of MRSA US300 clone amongst human clinical infections in St. Kitts [3]. Since the island is also home to a large monkey population, we recently applied a One Health approach to investigate staphylococcal diversity in vervet monkeys where we detected only methicillin resistant coagulase negative strains [4]. In our efforts to determine the role of invasive animals in antimicrobial resistance, we investigated the microbiomes of the small Indian mongoose on the island of St Kitts [5]. Mongooses have the typical microbiota of a carnivorous feeding pattern with a dominant abundance of Firmicutes (54.96%), followed by Proteobacteria (13.98%) and Fusobacteria (12.39%), and a relatively minor proportion of Actinobacteria (10.4%) and Bacteroidetes (6.40%). Some differences were observed based on the area where the animals were captured and between male and female animals [5]. While *S. aureus* is not a major component of the microbiome [5], we decided to further characterize the *S. aureus* isolates as there is a major problem with MRSA infections in humans on the island [3]. This One Health approach will allow us to determine potential reservoirs and determine the spread of strains in wildlife. Out of samples from the former study [5], we isolated 5 *S. aureus* and analyzed them by whole genome sequencing. Here we report the presence of a novel sequence type (ST) of methicillin susceptible *S. aureus* (MSSA) and MRSA ST5 in the intestines of mongooses in St Kitts.

## 2. Results

**Figure 1:**
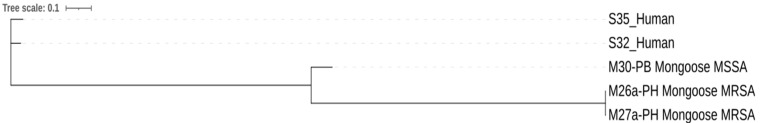
SNP tree of the 2 human MRSA and 3 mongoose *S. aureus* ST5 strains.

Out of 81 samples, five *S. aureus* isolates were obtained from five animals. Table 1 shows the details of the isolates. Two isolates were identified as MRSA and three as MSSA. Of the three MSSA, two belonged to a new sequence type, ST7434, as a single locus variant within the clonal complex 30, with spa type t019, while the third MSSA isolate was a ST5 t002. The two MRSA isolates, originating from two different animals caught in the same region (Paradise Heights, peri-urban area), were both ST5 spa type t002. The difference between both MRSA ST5 isolates was a single SNP. The two MSSA ST7434 isolates originated from different animals and different neighborhoods, but were very similar, showing only four SNPs difference. The difference between the MSSA ST5 and MRSA ST5 was substantial with about 524 SNPs difference. The ST5 MRSA were compared to two MRSA ST5, one spa type t002 and one t2235 isolated from human infections in St. Kitts [3]. The human strains differed from each other by 71 SNPs, and differed 530 SNPs from the closest mongoose strain, which was the MSSA ST5 (Figure 1).

The MSSA ST7434 had two exoenzymes, three genes of the gamma-hemolysin, six enterotoxins and the toxic shock syndrome toxin-1. Both the MSSA and MRSA ST5 strains, had substantially more virulence genes including host immune evasion genes and leucocidins. The virulence profile between the MRSA and MSSA ST5 strains were, however, not identical (Table 1).

In the MSSA strains, only the *blaZ* gene, encoding resistance against penicillin was detected and was associated with Tn552. The *blaZ* gene in strain M30-PB was present on a plasmid with rep20, together with the Superantigen enterotoxin SEA. BLAST search demonstrated that this is a common plasmid in staphylococci. In the MRSA strains; two *blaZ* genes were present, one in the SCC*mec* element and one on another location in the chromosome. The MRSA strains all carried the SCC*mec* type Iva (2B) and harbored resistance genes against macrolide antibiotics and aminoglycosides alongside the *mecA* gene as well as other plasmids (Table 1).

## 3. Discussion

This is the first description of *S. aureus* and MRSA isolates obtained from the small Indian mongoose (*Urva auropunctata*). Previously, MRSA has been isolated from captive *Cynenicillateillata* and *Helogale parvulaor* or the yellow and dwarf mongoose, respectively, both belonging to the same Herpestidae family. Those strains belong to the classical LA-MRSA CC398 and *mecC* carrying ST130 [6]. The captured mongooses were mainly from residential areas where they frequently roam among human garbage. In this study, the strains were isolated from fecal samples which is the most common staphylococcal carriage site. It remains to be determined what the prevalence in the nose or on the skin is. A larger study, including samples from different body sites in mongooses should be carried out to determine the actual prevalence and thus also the potential burden for human health on the island. Nevertheless, gastrointestinal carriage of MRSA in humans has been shown to be epidemiologically and clinically important [7,8]. The role of MRSA in the intestines of mongooses may have its importance in the general epidemiology and spread of MRSA at the human-wildlife interface.

MSSA were included in this study as we performed a non-selective isolation aim to investigate the diversity of *S. aureus* in the samples. Apart from a single MSSA ST5 we found two MSSA strains with a ST, ST7434, belonging to CC30, which is commonly found in humans but also in several animal species including marmots, camels, pigs, white stork and Portuguese buzzard [9]. It remains unclear whether ST7434 is specifically associated with mongooses as two very similar strains from different neighborhoods were isolated.

MRSA ST5 has previously been reported in animals [1], though not very often and is mainly found in swine in the US [10]. It is one of the most frequently detected MRSA STs in humans globally, especially in Asia, and has been mainly associated with SCCmecII [11,12]. Our strains, however, possessed the SCC*mec* type IVa (2B), which is the same cassette found in the predominant ST8 as well as the ST5 strains from St Kitts [3]. The strains were clonally highly related, but were isolated from different animals, but from the same geographical region, suggesting that this strain is spreading amongst mongooses or that the strain was picked up from a common source.

Since the mongoose MSSA strain, and not the mongoose MRSA strain, was the closest relative to the human MRSA ST5 strains, there is little indication for a direct transmission of MRSA between mongooses and humans. Nevertheless, host jumps of MSSA ST5 from humans to poultry has been described [13], though there is no information on potential host jumps from poultry to mongooses. The presence of MRSA in the poultry of St. Kitts is unknown and the current collection of ST5 strains from mongooses is also too limited to demonstrate potential relations with ST5 strains from other origins. We postulate however that the ST5 strains were originally from mongooses and acquired the methicillin resistance from potential human origin.

Of major relevance is the presence of the susceptible ST5 t002 strain. Since the strain differs substantially from the mongoose ST5 MRSA strains in SNPs, resistance genes, virulence genes, and plasmids, this leads to the assumption that the SCC*mec* element might have been acquired locally from human MRSA strains of St. Kitts. Further investigations isolating MSSA and MRSA from mongooses or other sources may bring clarity.

## 4. Materials and Methods

### 4.1. Sampling, Isolation and Identification

Eighty-one wild mongooses were collected between April and June 2017 in the federation of St Kitts and Nevis as previously described [5]. Briefly, mongooses were trapped in urban, peri-urban, and rural areas in live box traps (Tomahawk Live Trap, Wisconsin) to which they were attracted using bait. Trapped mongooses were transported to the Necropsy Laboratory and subsequently anesthetized with 3 mL vaporized isoflurane and euthanized via intra-cardiac injection of potassium chloride (1–2 mmol/kg). subsequently, fecal samples were collected from the rectum and distal part of the colon, aliquoted into sterile tubes and stored at −80 °C. Fecal samples were cultured on phenylethyl alcohol selective agar (Sigma-Aldrich, St Louis, MO, US) for the isolation of gram-positive bacteria. Isolates were purified and identified by MALDI-TOF MS (Biotyper, Bruker Daltonics, Germany) as previously described [4]. *S. aureus* was selected for further sequencing.

### 4.2. Sequencing and Sequence Analysis

For sequencing, overnight cultures were grown in tryptic soy broth at 37 °C with 200-rpm shaking. Genomic DNA from the staphylococcal strains was isolated using the DNeasy Blood and Tissue kit (Qiagen, Hilden, Germany). DNA purity and concentration were determined using the Nanodrop and Qubit instruments, respectively. Sequencing library preparation was accomplished using the Nextera XT kit and sequenced on a MiSeq using a paired-end 2 × 250 bp sequencing strategy, all following standard Illumina protocols (Illumina, Inc., San Diego, CA, USA).

Adapters were trimmed and assembled using unicycler and quality was assessed with QUAST using the Patric server (https://www.patricbrc.org/ (accessed on 1 September 2021). The following analyses were performed with pipelines from the Center for Genomic Epidemiology (http://www.genomicepidemiology.org/ (accessed on 1 October 2021): Kmer analysis to confirm the species identification (KmerFinder), ResFinder v.3.0 for the detection of resistance genes, PlasmidFinder v.2.0 for the detection of plasmid replicons, the SCCmec type was identified using SCCmecFinder, VirulenceFinder was used for the detection of virulence genes, MLST profiles were determined using “MLST” and spa type was determined using SpaTyper. Genomes were analyzed using CSIPhylogeny for Single Nucleotide Polymorphism (SNP) analysis. SNP tree was constructed using iTol (https://itol.embl.de (accessed on 15 October 2021). Strains were annotated using the RAST server with standard settings. Specific contigs were inspected manually and in some cases compared with other sequences using BLAST analysis. Plasmid fasta files were blasted for comparison to other plasmids in the databases. MLST profiles were submitted to pubMLST. Sequences are submitted to NCBI as PRJNA777776 with SRR16820787 to SRR16820793.

### 4.3. Ethics

This study was approved by the Ross University Institutional Animal Care and Use Committee under the IACUC Number 17.04.13.

## 5. Conclusions

MRSA ST5 t002 SCC*mec* IVa (2B) was isolated from wild invasive mongooses on a small Caribbean island. This reflects the broad host range of *S. aureus* and the potential reservoir of these clones that have been known to cause infections in humans. At the same time a new type of MSSA, ST7434, was present in mongooses, a single locus variant of CC30, commonly found in humans. Further studies are necessary to demonstrate the One Health aspect of MRSA in wildlife.

## Figures and Tables

**Table 1:** Characteristics of the isolated *S. aureus* strains.

Strain *	MLST	Spa	SCCmec	Resistance Genes	Virulence Genes	Plasmid Replicon Types
M2-WF	7434	t0019		*blaZ*	*hlgA, hlgB, hlgC,* *seg, sei, sem sen, seo, seu* *tst, aur, splE*	16, 5a
M21-FB	7434	t0019		*blaZ*	*hlgA, hlgB, hlgC,* *seg, sei, sem sen, seo, seu* *tst, aur, splE*	16, 5a
M26a-PH	5	t002	Iva(2B)	*blaZ-mecA-mph©-msr(A)-aph(3’)-III-aac(6’)-aph(2’’)*	*sak, scn* *hlgA, hlgB, hlgC, lukD, lukE* *seg, sei, sej, sem sen, seo, sep, ser seu* *aur, splA, splB*	5a, US70, 13, 21
M27a-PH	5	t002	IVa(2B)	*blaZ-mecA-©(C)-msr(A)-aph(3’)-III-aac(6’)-aph(2’’)*	*sak, scn* *hlgA, hlgB, hlgC, lukD, lukE* *seg, sei, sej, sem sen, seo, sep, ser seu* *aur, splA, splB*	5a, US70, 13, 21
M30-PB	5	t002		*blaZ*	*sak, scn* *hlgA, hlgB, hlgC, lukD, luKE* *sec3, sed, seg, sei, sej, sel*	20

* M: mongoose, animal number, location of trapping, FB: Frigate Bay, PH: Paradise Heights, PB: Potato Bay and WF: West Farm

## Data Availability

Sequences are submitted to NCBI as PRJNA777776 with SRR16820787 to SRR16820793.
